# Dwelling Characteristics Influence Indoor Temperature and May Pose Health Threats in LMICs

**DOI:** 10.5334/aogh.2938

**Published:** 2020-08-03

**Authors:** June Teare, Angela Mathee, Nisha Naicker, Cheryl Swanepoel, Thandi Kapwata, Yusentha Balakrishna, David Jean du Preez, Danielle A. Millar, Caradee Y. Wright

**Affiliations:** 1 Department of Environmental Health, School of Behavioural and Lifestyle Sciences, Faculty of Health Sciences, Nelson Mandela University, Port Elizabeth, SA; 2 Environment and Health Research Unit, South African Medical Research Council, Johannesburg, SA; 3 Department of Environmental Health, Faculty of Health Sciences, University of Johannesburg, Johannesburg, SA; 4 Epidemiology and Surveillance, National Institute of Occupational Health, Johannesburg, SA; 5 Biostatistics Unit, South African Medical Research Council, Durban, SA; 6 Department of Geography, Geoinformatics and Meteorology, University of Pretoria, Pretoria, SA; 7 Laboratoire de l’Atmosphère et des Cyclones, Université de La Réunion, Saint-Denis de La Réunion, FR; 8 Environment and Health Research Unit, South African Medical Research Council, Pretoria, SA

## Abstract

**Background:**

Shelter and safe housing is a basic human need that brings about a sense of ownership, self-sufficiency, and citizenship. Millions of people around the world live in inadequate dwellings in unhealthy areas, such as urban slums. These dwellings may experience indoor temperatures that impact inhabitants’ health. Indoor dwelling temperatures vary depending on many factors including geographic location, such as inland versus coastal. In an era of climate change, understanding how dwelling characteristics influence indoor temperature is important, especially in low- and middle-income countries, to protect health.

**Objective:**

To assess indoor temperature in low-cost dwellings located in a coastal setting in relation to dwelling characteristics.

**Methods:**

Indoor temperature and relative humidity loggers were installed from 1 June 2017 to 15 May 2018 in 50 dwellings in two settlements in a coastal town on the east coast of South Africa. Ambient outdoor temperature data were obtained from the national weather service, indoor temperature data were converted into apparent temperature, and heat index calculations were made to consider possible heat-health risks. A household questionnaire and dwelling observation assessment were administered. A mixed-effects linear regression model was constructed to consider the impact of dwelling characteristics on indoor apparent temperature.

**Findings:**

Among 17 dwellings with all data sets, indoor temperatures were consistently higher than, and well correlated (r = 0.92) with outdoor temperatures. Average differences in indoor and outdoor temperatures were about 4°C, with statistically significant differences in percentage difference of indoor/outdoor between seasons (p < 0.001). Heat indices for indoor temperatures were exceeded mostly in summer, thereby posing possible health risks. Dwellings with cement floors were statistically significantly cooler than any other floor type across all seasons.

**Conclusions:**

Low-cost dwellings experienced temperatures indoors higher than outdoor temperatures in part due to floor type. These results help inform interventions that consider housing and human health (n = 289).

## Introduction

Safe housing is a basic human need that contributes to a sense of belonging, ownership, identity, citizenship, and self-sufficiency [1]. Globally, 1.6 billion people live in inadequate housing, of which one billion reside in slums and informal settlements [2]. These settlements may be situated in environmentally unsafe or unhealthy areas, for example, in river flood zones and near industry. Many of the inhabitants who live there, live with little or no tenure for land or dwellings (e.g. rent informally) and typically have no or infrequent supply of basic services.

In addition, an important element of safe housing is thermal comfort. Cold and heat extreme temperatures impact human health. In general, people at increased risk of temperature-related illnesses are those with (1) pre-existing health conditions, such as cardiovascular conditions, respiratory conditions, psychiatric illness, alcohol/drug abuse, diabetes, hyper/hypotension, and so on, (2) extremes of age, (3) an inability to adapt their behaviour, such as Alzheimer patients, people who are confined to bed, and people with disabilities, (4) pregnant women, and (5) environmental challenges (such as people who reside on upper floors of buildings, dwellings with inadequate ventilation or lack of air conditioning in the home, people who live alone and care home residents) [3]. These environmental challenges are of particular concern given the environmental changes and climate change impacts being observed around the world [4].

Effects of indoor temperature on health implies that buildings are modifiers of the effect of weather and climate on health outcomes [3]. Some studies on these effects have been done in, among other countries, the United Kingdom (UK) [5] and the United States (US) [6]. However, further research is needed to understand indoor temperatures in relation to dwelling characteristics and how they relate to health in different climate zones in countries around the world, especially in low- and middle-income countries.

The World Health Organization (WHO) Housing and Health guidelines [7] have made conditional recommendations for the development and implementation of strategies to protect communities exposed to high ambient temperatures resulting in excess indoor heat. The relationship between indoor and outdoor temperature is non-linear with a strong correlation at warmer temperatures and a weak correlation at cold temperatures [8]. These differences may be due to warmer ‘cold’ temperatures indoors compared to outdoors during the cold season as dwellings may be insulated and/or people may be warming their dwellings, for example. Minimal risk and maximum acceptable indoor temperatures for heat-related health effects have been suggested. Indoor maximum acceptable temperatures range from 25°C (for the US and the UK) to 32°C (for Thailand). Positive correlations (but not always significant) have been established between indoor heat and sleep disorders, respiratory and cardiovascular disease, pregnancy outcomes, mental illness, blood pressure (both systolic and diastolic), general health, and body temperature [9,10,11,12,13,14,15,16,17]. For instance, significant correlations were for blood pressure [12] and body temperature [11]. There were some significant correlations for various pregnancy issues, but these were not carried through to final analyses [16].

Acclimatization is emphasised in the World Meteorological Organization-World Health Organization (WMO-WHO) guidelines [18], but this process may take several years to occur. While people may adapt to usual seasonal fluctuations in temperatures, they may not be able to adapt to variable temperatures and changes in temperature range. Unstable temperatures harm the cardiovascular and immune system and are associated with an increase in mortality [19,20]. Thermal insulation, housing location, building materials and house orientation, window shades, green spaces, ventilation, and air-conditioning can help to mitigate high indoor temperatures [7]. However, air-conditioning is not always an option due to increases in costs, energy consumption, and carbon emissions [21].

Current research in climate change suggests that the southern African region will experience more hot days and hotter heat waves for longer periods [22]. Heat waves that are unusual under present climate conditions will occur on a more frequent basis by 2040 [23]. Ambient temperatures in southern Africa may increase by a mean of 4.6°C by 2100 [24]. Warming in winter (June, July, and August months) was projected to be greater than warming in summer and spring, and the number of days during which indoor apparent temperature (‘real-feel’ metric for temperature) would be categorised as ‘potentially harmful’ will likely increase in the future [25].

Previous studies conducted in urban and rural areas in South Africa, which has multiple climatic zones, have found that household occupants are exposed to relatively high indoor temperatures in their dwellings, which could affect their health. An inland study in Giyani, a rural area in the north-east of South Africa, revealed that the sampled population was exposed to indoor daytime apparent temperatures exceeding 40°C in summer and 35°C in spring [25]. Another study in impoverished urban communities in Johannesburg found that during summer, indoor apparent temperatures in government-built, low-cost houses approached 35°C [26]. In that study, building materials such as carpets and cement ceilings increased indoor apparent temperatures. Scovronick and Armstrong [27]. investigated housing type impact on temperature-related mortality in the Eastern and Western Cape provinces. They considered broad housing types, that is, traditional, informal, formal low-cost, and so on, but they did not consider the dwelling characteristics in detail of each of these housing types. They found that future mortality burdens would be lower if informal housing was prioritised for replacement rather than traditional housing. These studies emphasize the vulnerability of rural and low-income populations to potentially adverse high indoor temperatures that may have impacts on their health and well-being.

Given the variation in indoor apparent temperature in relation to dwelling characteristics and geographic locations of communities located inland, the aim of this study was to assess indoor temperature in dwellings located in coastal settings and potential temperature-health risks while considering associated dwelling characteristics. While similar studies have been carried out in southern Africa (see above), this is the first study to focus on coastal communities. Communities living in coastal settings can face temperatures that are moderated by the ocean, a concern in the climate change context as sea surface temperature levels are rising [28]. Hence, understanding the temperatures experienced by communities in relation to dwelling characteristics living in both coastal and inland settings in Africa will help inform interventions and information for awareness campaigns and preparedness in the current and future changing climate.

## Data and Methods

### Study site and sample

**Figure 1:**
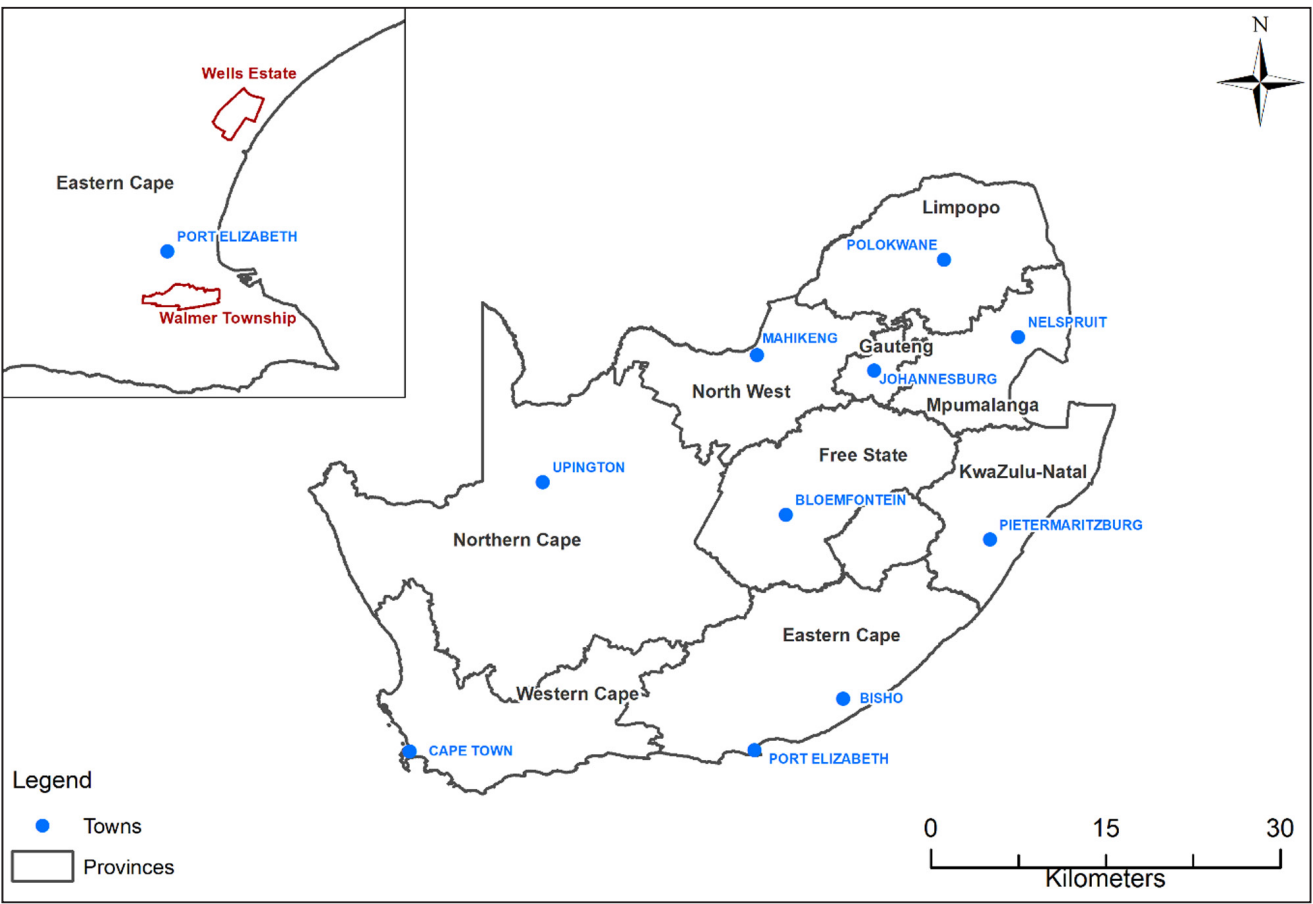
Location of Walmer Township and Wells Estate in the Eastern Cape Province of South Africa (Source: produced by the SAMRC).

The study was conducted in the Eastern Cape Province in the Nelson Mandela Bay area which serves as both a district municipality and a metropolitan municipality (Figure 1). The typical weather in Port Elizabeth is mild with austral summertime (December, January, February) and wintertime (June, July, August) minimum and maximum temperatures of 9°C and 20°C, and 17°C, and 27°C, respectively [29]. The town experiences winter rainfall with peaks of about 60 mm in August and September.

The Eastern Cape Province is known for being a poverty-stricken province and houses a total population of 6,562,053 [30]. About 49% of its population live in rural areas and 51% live in urban areas [30]. The province also has the highest proportion of poor residents with 41% of the total population living below the food poverty line of ZAR 441 (about USD 20) in 2015 [31].

**Figure 2:**
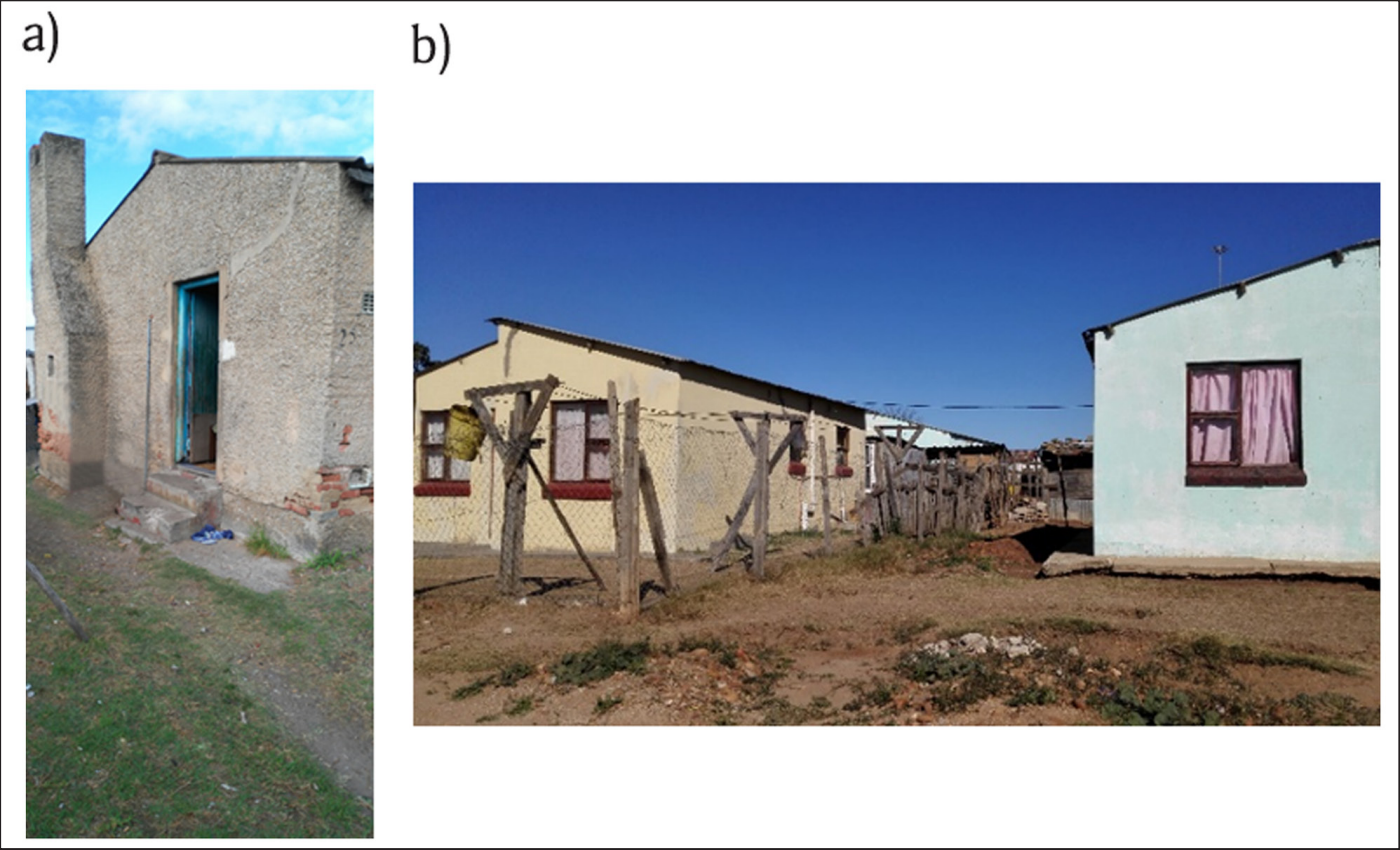
Examples of different dwelling types in **(a)** Walmer Township and **(b)** Wells Estate (Source: Captured by JT with permission from participants).

For this study, two areas were selected as they represented different environmental health exposure profiles for local communities in the Nelson Mandela Bay Municipal area. The areas were Walmer Township and Wells Estate. Walmer Township (also known as Gqebera) is an old, well-established neighbourhood situated within the city limits of Port Elizabeth and in close proximity to the International Airport. The housing includes a mix of low-cost houses, many in excess of 100 years of age (Figure 2). Wells Estate, situated approximately 20 km from Port Elizabeth City centre, was established in 2001 when low-cost houses were built in the area to house residents who had been displaced by the construction of the Coega Industrial Development Zone and the deep-water Port of Ngqura [32].

### Procedures for data collection

This study was carried out as part of the annual iBhayi Health and Environment study, which was a collaborative project undertaken by the South African Medical Research Council (SAMRC) and Nelson Mandela University (NMU). Research ethics clearance for the study was granted by the NMU (Certificate number: H14-HEA-ENV-001, 17 November 2014). Permission was sought from the Nelson Mandela Bay Municipality and local ward councillors in the study area.

Fifty houses in total were randomly selected from both areas and enrolled in the study for one year from 1 June 2017 to 15 May 2018. Permission to install a temperature and humidity logger, the Thermochron i-Button®, in the dwellings was obtained from a household respondent 18 years or older.

**Figure 3:**
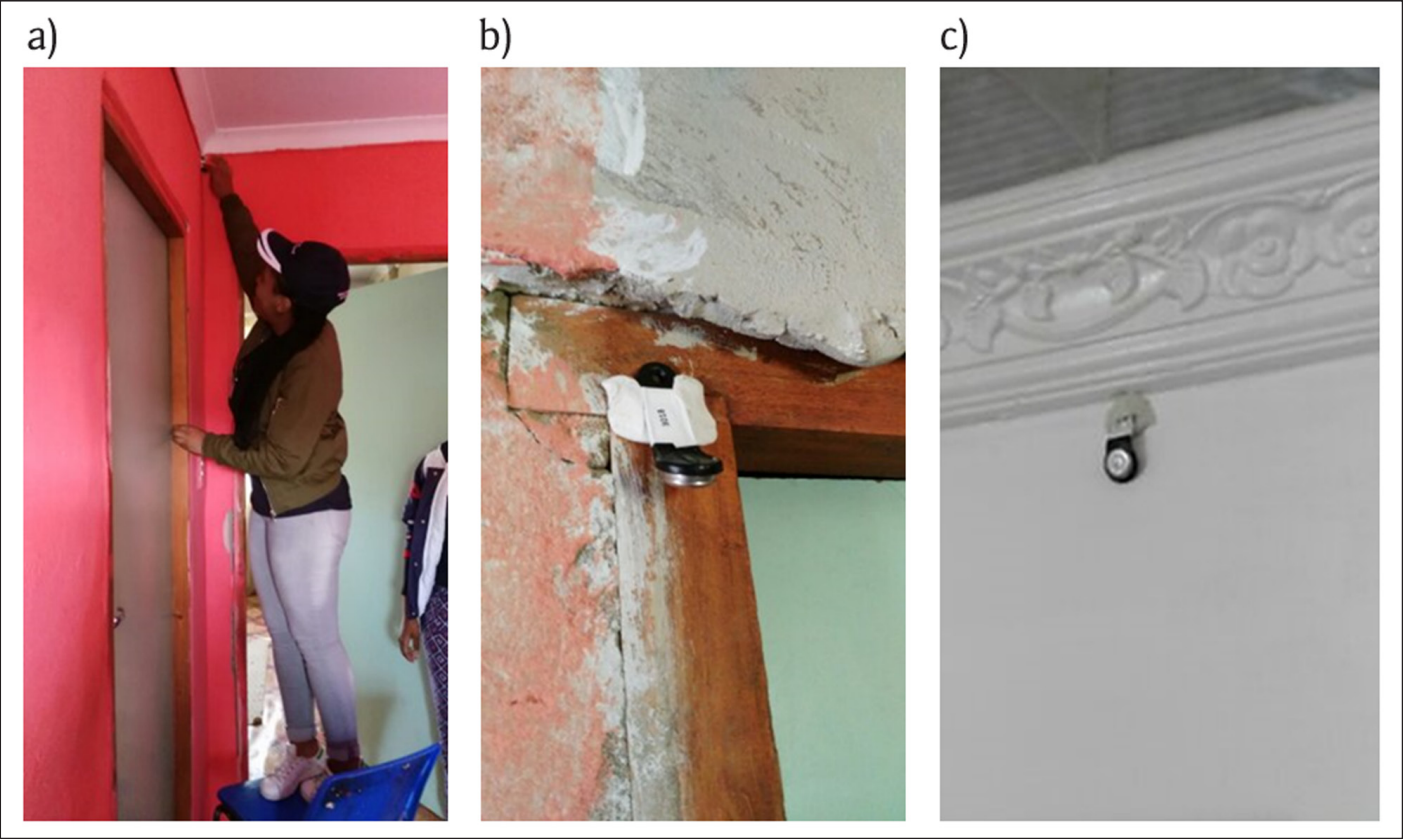
**(a)** Fieldworker installing an i-Button® temperature and humidity sensor and placement of i-Buttons® in some of the dwellings on **(b)** door frames and **(c)** walls (Source: Captured by JT with permission from participants).

All i-Button® loggers were tested in a controlled office environment prior to installation to ensure consistently correct data logging before being installed by trained fieldworkers (Figure 3).

The i-Buttons® were attached using putty adhesive and positioned away from cooking areas and heat-generating appliances, such as refrigerators. The typical dwellings in both study areas were approximately 50 m^2^ and majority of them comprised four spaces, two bedrooms, a lounge/kitchen and a bathroom with separate toilet. The i-buttons were placed in the lounge or the passage area outside the bedrooms approximately in the centre of the house. An installation information sheet was used to note the logger identity number, date and time of installation, location and position of logger, and confirmation of activation of each device to start taking measurements. The i-Buttons® were set to commence recording data from 1 June 2017 until their recovery in May 2018 during the 2018 iBhayi Health and Environment study. Six months after installation, a site inspection was carried out to confirm that the i-Buttons® were still in place and recording data. At the time, it was discovered that many i-Buttons® had been removed and collected by an unknown person.

The trained fieldworkers also recorded dwelling characteristics using an observation sheet and conducted a face-to-face questionnaire with a household respondent from whom consent for participation in the study was obtained (see above).

### Indoor temperature and humidity logger

**Figure 4:**
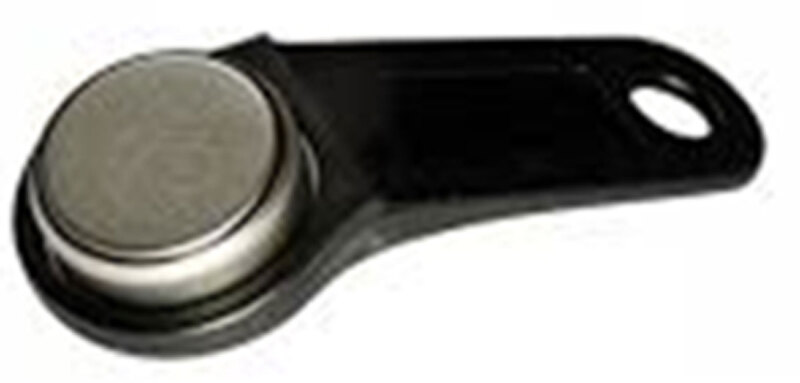
Thermochron i-Button® DS1923 temperature and humidity logger (Source: From Manufacturer).

The Thermochron i-Button® DS1923 temperature and humidity logger (Figure 4) supplied by Fairbridge Technologies (Johannesburg, South Africa) is a temperature device the size of a small disc battery (17 mm in diameter and 6 mm thick). Calibration of all devices was performed by the manufacturer. The device is held in a specially designed plastic fob which is a black plastic holder. Each device was marked with a unique serial number and was programmed using the software supplied by the manufacturer. The logger was set to record every five minutes continuously throughout the study.

### Outdoor Temperature Data

Meteorological data were collected from the South African Weather Service (SAWS) station at the Port Elizabeth International Airport (33.98°S, 25.62°E) which is located less than 5 km from Walmer Township and approximately 15 km from Wells Estate. The station is located 60 m above sea level and experiences an annual mean minimum and maximum temperature of 14°C and 22°C, respectively [33]. Hourly measurements of temperature and humidity were obtained for the period from June 2017 to May 2018. From these data the daily average temperature was calculated.

### Heat index calculations

In addition to measured indoor and outdoor temperature, we calculated heat index (Equation 1) using indoor and outdoor temperature and relative humidity measurements [34]. Heat index has been used widely in environmental health studies as a measure of heat exposure because it combines the effect of different weather factors on the human body [35,36]. The U.S. National Weather Service (NWS) uses a heat index table developed by the U.S. National Oceanographic and Atmospheric Administration (NOAA) to issue heat advisories (Table 1). Similar to Quinn et al., [37] the heat index calculations for the study days were used for comparison to the possible heat impacts described in Table 1 because no African classification exists. Heat index ranges have been assigned potential health risks on the body and labelled for ‘caution’, ‘extreme’, ‘danger’, and ‘extreme danger’. Health effects include, for example, in the ‘caution’ (26°C–32°C) range, fatigue being possible with prolonged exposure and/or physical activity, and even heat stroke, as in the ‘extreme danger’ classification (≥54°C).

Equation 1 \begin{array}{l}
HI = 0.5*\left({T + 61.0 + [(T - 68.0)*1.2]} \right.\\
\;\;\;\;\;\;\;\;\left. { + \;(RH*0.094)} \right)
\end{array}

Where *T* = measured ambient or indoor temperature (°C)

*RH* = relative humidity (%).

**Table 1:** Potential health impacts for four heat index threshold ranges.

Heat index (°C)	Risk level	Effect on Body

27–32	Caution	Fatigue and discomfort possible with prolonged exposure and/or physical activity
32–41	Extreme caution	Heat stroke, sun stroke, heat cramps, or heat exhaustion possible with prolonged exposure and/or physical activity
41–54	Danger	Heat cramps or heat exhaustion likely, and heat stroke and sun stroke possible with prolonged exposure and/or physical activity
>54	Extreme danger	Heat stroke, sun stroke highly likely

### Household questionnaire

The questionnaire was similar to the questionnaire applied in the Johannesburg Health, Environment and Development (HEAD) study [26]. Data from sections on socio-demographics, housing, and neighbourhood were included here. The socio-demographic section requested information about country and province of birth, number of household members and dwellings accommodating the household on the plot of land, age, gender, and education of all household members, main household language, length of stay in dwelling, monthly household income, and access to medical aid, and possession of household items.

The housing and neighbourhood section captured the type of dwelling, age of dwelling, number of rooms in the dwelling, and dwelling characteristics such as peeling paint, cracks in walls, ventilation, lighting, broken windows, leaks, mould, dampness, overcrowding, and dust. Questions on thermal comfort asked whether the house is uncomfortably hot or cold and how to ameliorate these conditions in summer and winter, respectively. Fuel use for cooking and heating as well as presence of pets and environmental tobacco smoke in the dwelling were also recorded.

### Dwelling observations

The dwelling observation sheet was used to capture data by the trained fieldworker about the dwelling. This included the type of roof (roof material, colour, shape), ceiling (none, cement, wood, board), walls (plastered brick, brick, wood, metal sheeting, stone, internal versus external), windows (number, blind, awning, operational, type of glass), floors (cement, linoleum, wood, brick, tiles, sand), methods used for temperature control (portable fan, ceiling fan, air conditioning), and presence of shade trees (yes/no, number).

### Statistical analyses

All statistical analyses were performed in STATA version 15 (StataCorp, TX, USA). Data recorded by the iButton® loggers were linked to household questionnaire data and dwelling observation data using a unique household identifier number. First, linear regression and Pearson’s correlation was used to assess the association between indoor temperature (dependent variable) and SAWS outdoor temperature. Differences in temperature (by °C and by percentage difference) were calculated between indoor and outdoor temperature using the following Equation (2):

Equation 2 PD = 100\;\, \times \,\;\frac{{\left[ {in - out} \right]}}{{\frac{{\left({in - out} \right)}}{2}}}

Where PD is percentage difference, *out* refers to outdoor temperature and *in* refers to indoor temperature [38].

Second, to consider the relationships between indoor apparent temperatures and dwelling characteristics where heat index was calculated for the indoor dwellings using Equation (1). Results were considered to evaluate possible health risks according to Table 1, as well as to consider the impact of dwelling characteristics, such as ceiling material, on indoor apparent temperature. A mixed-effects linear regression model with a random intercept and slope for each dwelling to control for the lack of independence between measurements from the same dwelling was applied. Results were considered statistically significant for p-values less than 0.05.

## Results

### Sample description

Of the 50 iButton loggers installed, 25 per settlement, only 27 loggers were retrieved from the two areas; 11 sensors from Walmer Township and 16 sensors from Wells Estate. After data quality control, including matching indoor temperature data with dwelling characteristics and household questionnaire data, there were 22 households available for inclusion in the analyses. Of the 22 households, 17 had complete information for a year of indoor temperature measurements as well as dwelling characteristics.

### Indoor versus outdoor temperatures

**Figure 5:**
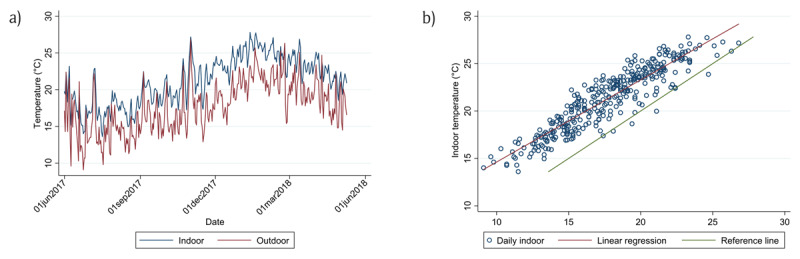
**(a)** Time series of daily average dwelling indoor temperatures for 27 dwellings compared to SAWS daily average outdoor temperatures. **(b)** Linear regression for the study period between the average daily indoor temperatures for all dwellings and daily SAWS average outdoor temperature (intercept β_0_ = 5.96, SE(β_0_) = 0.38, slope β_1_ = 0.87, SE(β_1_) = 0.02, model R^2^ = 0.84, correlation r = 0.92). The green reference line (slope = 1) indicates when dwelling indoor temperatures were equal to the SAWS daily average outdoor temperatures.

Figure 5a shows the daily average indoor temperature measurements of all the dwellings in relation to the SAWS daily average outdoor temperatures. The indoor temperatures were consistently higher than the outdoor temperatures. This was most evident from October 2017 to April 2018. Furthermore, there was a strong linear relationship between the indoor and outdoor temperatures (r = 0.92) (Figure 5b).

**Figure 6:**
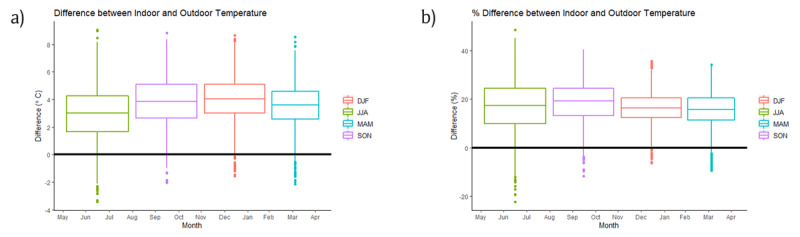
Boxplots of the difference between indoor and outdoor temperatures: Line represents the median, whiskers represent scores outside the middle 50%, box length represents interquartile range. **(a)** Temperature difference represented in degrees Celsius **(b)** Temperature difference represented as a percentage of indoor temperature.(Note: Summer DJF: December, January, February = summer; Winter JJA: June, July, August = winter; Autumn MAM: March, April, May = autumn; Spring SON: September, October, November = spring).

Figure 6a shows that variability in actual temperature was greatest during winter (June, July, August in green). Average differences in indoors and outdoors temperatures were about 4°C (a little less at about 3°C during winter). The figure also shows that during all seasons, indoor temperatures were on average higher than outdoors temperatures. The percentage difference between indoor and outdoor temperatures illustrates the range in temperature difference between houses across seasons (Figure 6b). While the percentage difference was similar for all seasons (about 20%) the percentage difference ranges were different: wide for winter and tighter for summer (similar to autumn). The differences between the seasons, both in degrees Celsius and percentage were statistically significant (p < 0.001 for both).

**Figure 7:**
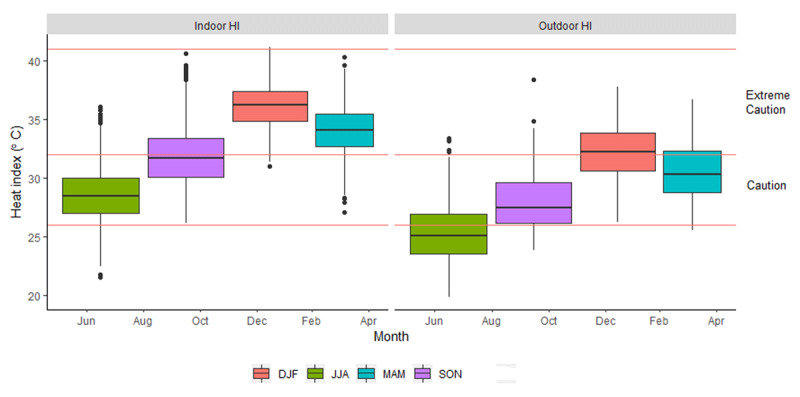
Boxplot of heat index for indoors and outdoors according to the health risk index.

Figure 7 shows the boxplots of indoor and outdoor daily heat index values. The potential health risks, as explained in Table 1, are indicated on the figures as red lines. Mean indoor heat index values were generally higher than outdoors across all seasons. The highest peak of heat index, 41°C, was indoors during summer. During summer and autumn, indoor heat index values were in the extreme caution risk level ranging between 31°C–41°C and 27°C–40°C respectively. Therefore, household occupants had an increased risk of experiencing heat cramps and heat exhaustion. During winter, mean outdoor heat index posed no health risk; however, mean indoor heat index was sometimes in the caution range (28°C) exposing inhabitants to possible fatigue and continued exposure could result in heat cramps.

### Dwelling characteristics and indoor apparent temperatures

The majority of dwellings were reportedly built by a professional builder, were more than 15 years old, and had dwellings comprising more than one room (Table 2). Presence of indoor environmental tobacco smoke and pets in the home were reportedly low. All households used electricity for cooking and heating water.

**Table 2:** Self-reported socio-demographic and dwelling characteristics in the study population for Walmer Township (n = 9) and Wells Estate (n = 13).

Characteristic	Walmer	Wells	Total	

	n	n	n	%

**Gender of respondent**				
Male	2	3	5	23
Female	7	10	17	77
How many people, including you, make up the main household? (mean (SD))	4 (2)	4 (1)	4	2
How many children under five years of age are part of this household?	2	4	6	–
**How long has this household been living in this dwelling?**				
Months (one year or less)	0	1	1	5
One year or more	9	12	21	95
**Type of dwelling used by household**				
Formal house built by professional builder	8	12	20	91
Formal house that was self-built	1	1	2	9
Approximately how old in years is the dwelling? (median, (IQR))	30 (15–60)	15 (15–16)	16 (15–30)	–
**In this dwelling, is there a**:				
Kitchen (for cooking only)	8	13	21	95
Bathrooms/toilets	6	12	18	82
Dining rooms	5	10	15	68
Lounge	3	3	6	27
Bedrooms	9	13	22	100
One main room	0	2	2	9
**What do you use mainly for indoor heating?**				
None	3	5	8	36
Electricity	5	7	12	55
Paraffin	1	1	2	9
Gas	0	0	0	–
Wood	0	0	0	–
Coal	0	0	0	–
Imbhawula (burn bottom-up)	0	0	0	–
Other	0	0	0	–
**Are any pets or animals kept inside the house?**				
Yes	0	4	4	18
No	9	9	18	82
**What do indoor household smokers smoke?**				
Cigarettes	2	4	6	27
Pipe tobacco	0	0	0	–
Hookah/hubby bubbly	0	0	0	–
Electronic cigarettes	0	0	0	–
Other	0	0	0	–

Characteristics of main dwellings observed by fieldworkers showed that most houses had roofs made from metal sheeting and had ceilings made from boards. Interior and exterior walls were plastered bricks, and floors were mostly either tiled or made from linoleum (Table 3). Natural temperature control by opening door(s) and window(s) prevailed (>80%) compared to use of portable fans (nobody had access to air conditioning), for example and few houses had shade trees nearby their dwelling (n = 6).

**Table 3:** Dwelling observations of main dwellings as observed by trained fieldworkers in the study population for Walmer Township (n = 9) and Wells Estate (n = 13).

Dwelling Characteristic	Walmer	Wells	Total	

	n	n	n	%

**Roofing material used**				
Clay tiles	1	0	1	5
Metal sheeting	8	13	21	95
Concrete	0	0	0	–
Other	0	0	0	–
**Shape of roof**				
Gable	8	8	16	73
Flat	1	4	5	23
Other	0	0	0	–
*Missing*	0	1	1	4
**Ceiling material used**				
No ceiling	2	0	2	9
Cement	0	0	0	–
Wood	1	0	1	5
Ceiling boards	6	13	19	86
Other	0	0	0	–
**Exterior walls**				
Plastered brick	7	11	18	82
Brick	2	2	4	18
Wood	0	0	0	–
Metal sheeting	0	0	0	–
Stone	0	0	0	–
Other	0	0	0	–
**Interior walls**				
Plastered brick	7	11	18	82
Brick	2	2	4	18
Wood	0	0	0	–
Metal sheeting	0	0	0	–
Stone	0	0	0	–
Other	0	0	0	–
**How many windows are there in the dwelling?**				
1 to 3	1	1	2	9
4 to 6	7	10	17	77
>6	1	0	1	5
*Missing*	0	2	2	9
**Do the windows have shade protection inside or outside (e.g. blinds, awnings, etc.)?**				
Yes	7	7	14	63
No	2	5	7	32
Some	0	0	0	–
*Missing*	0	1	1	5
**Can the windows be opened?**				
Yes	9	11	20	90
No	0	1	1	5
*Missing*	0	1	1	5
**How would you describe ventilation in this dwelling**:				
No problem	8	9	17	77
Moderate problem	1	3	4	18
Major problem	0	1	1	5
**How would you describe mould in this dwelling**:				
No problem	4	4	8	36
Moderate problem	2	7	9	41
Major problem	3	2	5	23
**Flooring material used**				
Cement	1	2	3	14
Linoleum	3	4	7	32
Wood	1	3	4	18
Brick	0	0	0	–
Tile	5	5	10	45
Earth	0	0	0	–
Other	0	0	0	–
**Temperature control**				
Yes	1	3	4	18
No	8	10	18	82
**If yes, please indicate method used**				
Portable fan	1	1	2	9
Ceiling fan	0	0	0	–
Air conditioner	0	0	0	–
Other	0	2	2	9
**Are there shade trees close to the dwelling?**				
Yes	4	2	6	27
No	5	11	16	73

In relation to dwelling characteristics, the only variable with a relationship to indoor apparent temperature was the floor type of the dwelling (Table 4). Dwellings with cement floors were cooler than dwellings with any other type of floor, that is linoleum, wood, and tiles.

**Table 4:** Coefficients (95% Confidence Intervals [CIs]) from the mixed-effects regression of indoor apparent temperature and dwelling characteristics. The model was adjusted for clustering by dwelling.

Dwelling characteristics	Indoor apparent temperature (°C) n = 17		

	Coefficient	95% CIs

**Roof type**			
Gable^#^	0	
Flat	–0.09	–0.87–0.69
**Roof material**			
Clay tile^#^	0	
Metal sheeting	0.53	–1.63–2.70
Concrete	–1.07	–4.06–1.91
**Floor**			
Cement^#^	0	0.19–2.72*
Linoleum	1.45	1.18–3.86*
Wood	2.52	0.44–3.63*
Tiles	2.04	0.41–3.18*
Other, e.g. dung	1.80	
**Exterior**			
Plastered brick^#^	0	
Brick	0.85	–0.59–2.30
**Shade trees**			
No#	0	
Yes	0.20	–0.48–0.89
**Model constant**	21.53	18.46–24.61*

*Note*: * p < 0.05; # indicates the reference variable; CI indicates confidence interval.

## Discussion

Improved housing conditions are key to meet the targets of Sustainable Development Goal (SDG) 11 (Sustainable Cities and Communities) and SDG 3 (Good Health and Well-Being), among others. This is especially so in a climate change context, where some parts of the world will experience warming and other parts cooling, or a combination of both at different times of the year [39]. This study aimed to assess indoor temperatures in low-income dwellings located in a coastal setting in relation to relevant dwelling characteristics. Improving our understanding of temperatures experienced by communities living in different locations, such as coastal and inland settings, in southern Africa will help inform interventions and strategies for healthy housing interventions and development.

In our study, indoor temperatures ranged between about 14°C–27°C with the lowest indoor temperatures experienced during winter. These temperatures are similar to the indoor dwelling temperature recommendations for dwellings in sub-tropical countries, such as South Africa, of between 18°C–25°C [40]. The indoor temperatures of the dwellings observed in this study were on average 4°C higher compared to the outdoor temperatures all year round. There was also a strong correlation between indoor and outdoor temperature, stronger than the correlation between the same variables in the study by Naicker et al [26]. conducted in urban inland dwellings. This may suggest that outdoor temperatures may be a better indicator of indoor temperatures at coastal sites compared to inland sites.

The risk for potential heat-related health impacts was greater indoors compared to outdoors. During summer and autumn, indoor heat index temperatures were within the extreme caution risk level. Indoor heat index was higher indoors than outdoors across all the seasons, therefore being indoors did not provide residents relief from heat exposure. A previous study in New York, USA, used the same definition of a heat index that was used in this study as an exposure metric to understand the effect of temperature on mortality [41]. They found that the temperature–mortality relationship persisted for several days and heat index between 35°C and 38°C was associated with higher mortality during the following three days [41]. Another study evaluated the association between heat index and morbidity and mortality in New England, USA [42]. Their results showed that heat index of 35°C was associated with a cumulative 7.5% (95% CI: 6.5%, 8.5%) higher rate of emergency department visits and death compared to a 5.1% (95% CI: 0.2%, 10.3%) increase when heat index was 24°C [42]. These studies show that the communities in this study face health impacts due to exposure to elevated indoor temperatures, especially during summer when mean heat index exceeded 35°C.

The only dwelling characteristics associated with indoor apparent temperature was having a cement floor. Findings showed an increased likelihood of warmer temperatures indoors in dwellings with tiles, wood, linoleum, and other flooring types compared to those dwellings that had cement floors. In contrast, in a study using model houses built in the Western Cape, concrete flooring led to indoor maximum temperatures being greater than the maximum outdoor temperatures [40]. However, this study was a modelling study in a passive solar, energy efficient house, hence dissimilar from all of the dwellings in our study. While cool concrete floors during summer may bring some relief from high indoor temperatures, concrete floors also show greatest percentage temperature difference compared to other floor types during wintertime. This may pose a risk for exposure to cold temperatures during winter. Temperatures in this study did not fall below 0°C; however, communities living in inland areas where temperatures regularly decrease below 0°C during winter may see even large percentage differences between indoor and outdoor temperature in winter in dwellings with concrete floors. Given the relatively small temperature difference between indoor and outdoor temperatures found in the current study, concrete floors may be appropriate, and should be earmarked for further investigation in coastal settings. However, alternative flooring to concrete may be better suited to inland dwellings where winter outdoor temperatures are cooler than coastal temperatures. This information is important since all government-provided housing in South Africa, regardless of the location, uses concrete flooring [43].

It has been calculated that elevated temperatures are associated with around 290,000 deaths (or 3.4% of the national burden of disease) in South Africa annually [44]. Should further studies with increased statistical power confirm the association found in the current study between cement flooring and lower indoor temperature, a benefit may apply to nearly 4 million South African households living in low cost (RDP) housing units, a significant proportion of which is fitted with cement flooring. On a large scale, cement flooring installed in RDP or government-subsidized housing, may be contributing to a reduction in temperature-related mortality. Alongside other housing and public health measures, relatively low-cost cement flooring should be assessed for its contribution to cooler indoor temperatures and lowered mortality during warmer months, but weighed against its contribution to mortality during cold weather [44].

Several limitations restrict generalisation of these study results. The sample size was relatively small due to the loss of temperature loggers. The comparison of indoor to outdoor temperature data from the SAWS data may have been influenced by the distance between the SAWS station and Wells Estate, which was farther away from the SAWS station than Walmer Township. We could have overcome this by installing commercially available weather stations in each of the towns and preferably more than one in each town to monitor differences in temperature, wind speed, and direction, and so on. Future studies should aim for larger sample sizes and include detailed information on the orientation of the house in relation to North/South since this can influence indoor temperature, among other variables such as roof colour, presence of awnings and outdoor shade nearby the dwelling [45]. While observations were made by fieldworkers regarding whether there appeared to be a problem in a dwelling – ventilation, presence of mould, etc. – a more useful question would have been whether dwelling habitants ventilated their homes daily (i.e. opened doors and windows, and preferably responses for all four seasons). Natural ventilation was reportedly used by about 80% of the study participants. Natural ventilation only marginally reduces risk of dwellings overheating in subtropical and tropical areas [46]. Another research step should be to analyse in detail winter temperatures experienced by inhabitants of dwellings in relation to heat index and potential health impacts at each risk level.

## Conclusions

Expanding knowledge on indoor dwelling temperatures versus outdoor ambient temperatures in communities set in different geographic locations is important for crafting nuanced advice around healthy housing construction materials and design. Here, occupants of coastal dwellings faced challenges around maintaining thermal comfort in their homes, especially during winter but also during transitional seasons, with floor type, amongst other factors, possibly playing a role. Some dwellings still experienced indoor heat index levels exceeding outdoor ambient heat index leading to potential health risks for inhabitants including increased risk of morbidity and mortality, particularly among vulnerable groups. Such observations are important and can be used to inform the development and implementation of policies and interventions around indoor housing temperatures, in this case heat and health, in low-income communities in LMICs.
